# Clostridium difficile infection in hospitalized patients with inflammatory bowel disease: Prevalence, risk factors, and prognosis: Erratum

**DOI:** 10.1097/MD.0000000000010023

**Published:** 2018-02-23

In the article, “Clostridium difficile infection in hospitalized patients with inflammatory bowel disease: Prevalence, risk factors, and prognosis”,^[1]^ which appeared in Volume 97, Issue 5 of *Medicine*, the authors want it noted that Drs Keren Hod and Iris Dotan contributed equally to the article.

## References

[1] Maharshak N Barzilay I Zinger H Clostridium difficile infection in hospitalized patients with inflammatory bowel disease: Prevalence, risk factors, and prognosis. *Medicine*. 2018 97:e9772. 29384868 10.1097/MD.0000000000009772 PMC5805440

